# Response to Collinson et al. Comment

**DOI:** 10.1097/HS9.0000000000000491

**Published:** 2020-10-26

**Authors:** Miriam Belmonte, Nina F. Øbro, David G. Kent

**Affiliations:** 1 Wellcome MRC Cambridge Stem Cell Institute, University of Cambridge, Hills Road, Cambridge, United Kingdom; 2 Department of Hematology, University of Cambridge, United Kingdom; 3 Department of Clinical Immunology, Copenhagen University Hospital, Rigshospitalet, Copenhagen, Denmark; 4 York Biomedical Research Institute, Department of Biology, University of York, York, United Kingdom.

In response to our recent paper which identified GRO-α, EGF and Eotaxin as potential biomarkers in chronic phase myeloproliferative neoplasms,^1^ Collinson et al explored one of the possible cellular sources of these molecules by measuring levels of transcripts produced (and stored) within platelet granules.^2^ Mutant megakaryocytes and platelets have been previously implicated in MPN pathogenesis, including in the fibrotic processes which is associated with poor prognosis.^3,4^ In line with our data, high EGF transcript levels were observed in platelets from essential thrombocythemia (ET) and polycythemia vera (PV) patients. On the other hand, GRO-α transcript levels in platelets were highest in myelofibrosis (MF) patients whereas our study reported that high GRO-α was present in chronic phase patients who eventually transformed to MF in samples taken prior to treatment initiation. Assuming that the Collinson et al. samples were also collected prior to treatment, there are several possible explanations for this discrepancy. First, it could be due to transcript levels not mirroring protein levels, as has been described previously in hematopoietic cells.^5^ Second, while the relative amounts of transcript might be highest in platelets, it is possible that the bulk of GRO-α is produced by other cell types, thereby minimizing the impact of relative changes in one cellular fraction. Finally, as suggested by the authors, platelet “exhaustion” or failed production in MF patients could contribute to differences. Irrespective of the underlying reason, the Collinson et al report emphasizes the importance of characterizing the sources of GRO-α in order to identify the arbiters of the inflammatory cytokine phenotype observed in numerous MPNs. Antibody stains in tissue sections, spatial transcriptomic analysis,^6^ or further proteomic profiling might help resolve the cellular drivers of disease.

Overall, this study extends our examination of the cellular drivers responsible for different cytokine levels across MPN subtypes, surveying platelets as a potential mediator of these differences. It is exciting to see additional research in this space which reaches beyond the simple identification of potential biomarkers to try and understand the cellular sources of disease modulators. Such studies could reveal distinct mechanisms of pathogenesis across different disease subtypes or throughout disease progression. It is already clear that cytokines are diverse across patients and there is accumulating evidence that they clarify some aspects of the disease heterogeneity that cannot be explained by genetic mutations alone.
